# Implementation strategies to increase access and demand of long-lasting insecticidal nets: a before-and-after study and scale-up process in Mozambique

**DOI:** 10.1186/s12936-017-2086-3

**Published:** 2017-10-25

**Authors:** Jorge A. H. Arroz, Chandana Mendis, Liliana Pinto, Baltazar Candrinho, João Pinto, Maria do Rosário O. Martins

**Affiliations:** 1 World Vision International, Maputo, Mozambique; 2 National Malaria Control Programme, Maputo, Mozambique; 3 Global Health and Tropical Medicine, GHTM, Instituto de Higiene e Medicina Tropical, IHMT, Universidade Nova de Lisboa, UNL, Rua da Junqueira 100, 1349-008 Lisbon, Portugal

**Keywords:** Before-and-after design, Implementation strategies, Implementation study, Long-lasting insecticidal nets, Universal coverage bed nets campaign, Mozambique

## Abstract

**Background:**

The universal coverage bed nets campaign is a proven health intervention promoting increased access, ownership, and use of bed nets to reduce malaria burden. This article describes the intervention and implementation strategies that Mozambique carried out recently in order to improve access and increase demand for long-lasting insecticidal nets (LLINs).

**Methods:**

A before-and-after study with a control group was used during Stage I of the implementation process. The following strategies were tested in Stage I: (1) use of coupons during household registration; (2) use of stickers to identify the registered households; (3) new LLIN ascription formula (one LLIN for every two people). In Stage II, the following additional strategies were implemented: (4) mapping and micro-planning; (5) training; and (6) supervision. Odds ratio (OR) and 95% confidence interval (CI) were used to compare and establish differences between intervened and control districts in Stage I. Main outcomes were: percentage of LLINs distributed, percentage of target households benefited.

**Results:**

In Stage I, 87.8% (302,648) of planned LLINs were distributed in the intervention districts compared to 77.1% (219,613) in the control districts [OR: 2.14 (95% CI 2.11–2.16)]. Stage I results also showed that 80.6% (110,453) of households received at least one LLIN in the intervention districts compared to 72.8% (87,636) in the control districts [OR: 1.56 (95% CI 1.53–1.59)]. In Stage II, 98.4% (3,536,839) of the allocated LLINs were delivered, covering 98.6% (1,353,827) of the registered households.

**Conclusions:**

Stage I results achieved better LLINs and household coverage in districts with the newly implemented strategies. The results of stage II were also encouraging. Additional strategies adaptation is required for a wide-country LLIN campaign.

**Electronic supplementary material:**

The online version of this article (10.1186/s12936-017-2086-3) contains supplementary material, which is available to authorized users.

## Background

In 2015, an estimated 212 million cases of malaria occurred worldwide [1]. Most of the cases in 2015 were in the African Region (90%) [1]. In the same year, it was estimated that there were 429,000 deaths from malaria globally with an estimated 92% of deaths occurring in the African region [1]. In Mozambique, malaria is a high burden disease, with a prevalence of 40.2% in children between 6 and 59 months of age [2]. The effective use of long-lasting insecticidal nets (LLINs) can reduce all-cause child mortality (by 22%) and malaria morbidity [3, 4], and is also associated with a community-wide decrease in malaria transmission [5]. The current challenge is to ensure access and ownership of LLINs in order for at least 80% of the population (and households) to be covered, and make appropriate use of them [6]. Universal coverage campaigns (hereinafter referred to as UCC) are widely proven to be a health intervention that can rapidly overcome the low LLIN access and ownership coverage [7], and are the most cost-effective malaria intervention [8, 9].

In 2015 66% of households in Mozambique had at least one LLIN, and 39% of households had one LLIN for each two persons [2]. These figures are considered as low coverage when compared with the target of at least 80% of households with sufficient LLINs to achieve universal coverage (i.e. one LLIN for every two persons) [6]. Therefore, implementation strategies were put in place in campaigns to improve LLIN distribution and reach the desired targets. These strategies were based on the findings that campaigns that make use of vouchers or coupons have better LLIN ownership outcomes [10–15].

This study reports the intervention and implementation strategies that Mozambique carried out in order to improve access and increase demand for LLIN use. A set of implementation strategies is described over two distinct stages: Stage I—a small pilot study in two rural districts; Stage II—a massive pilot in one northeast Mozambican province. The following research questions were addressed: are implementation outcomes changing with the new strategies compared with the old strategies? Which lessons can be learned from the implementation process?

## Methods

### Study design

Stage I was undertaken between October and December 2015. Using a before-after design, the pilot was carried out in four rural districts:

- Intervention districts: Gurue (intervention 1) and Sussundenga (intervention 2), in Zambezia and Manica provinces, respectively;
- Control districts: Alto-Molocue (control 1) and Machaze (control 2), also in Zambezia and Manica provinces, respectively.

This pilot tested a few innovations and adaptations of the model in place for UCC, namely: using coupons to do household registration, using stickers to identify registered households, and a new LLIN ascription criterion—one LLIN for every two persons. These innovations and adaptations led to higher coverage of outcome indicators in Stage I (see results section), guiding a massive pilot of the intervention and strategies in Stage II.

Stage II was carried out in the 23 districts of Nampula province (a northeast province of Mozambique, and the most populated of the country). During this stage complementary components of the strategy were added in order to improve planning and implementation processes. This was done with the technical support of Alliance for Malaria Prevention (AMP). Lessons learned from this massive pilot were used to better understand implementation challenges, and guide the country-wide UCC.

### Rationale of selection

**Fig. 1:**
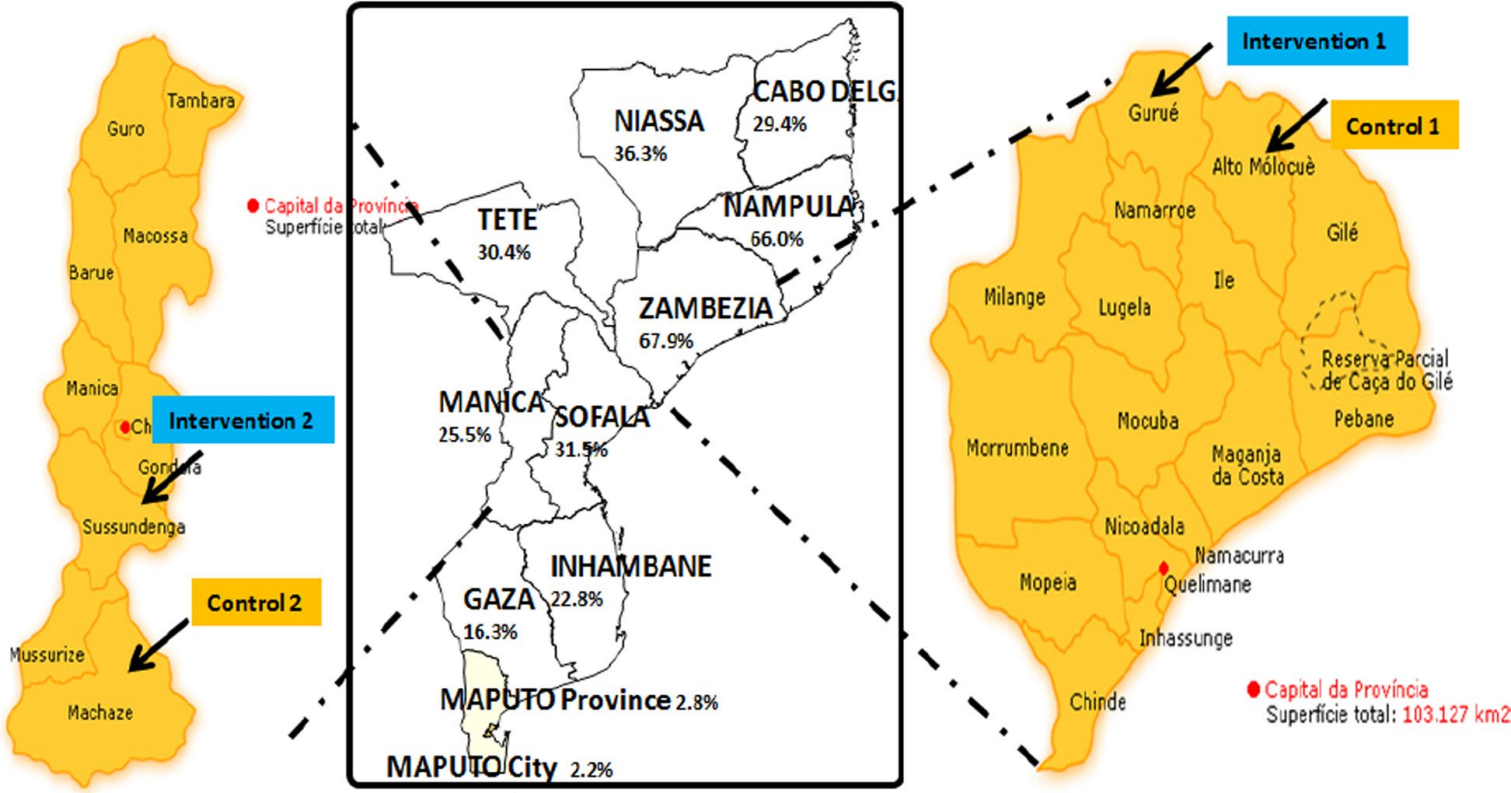
Malaria prevalence in Mozambique and intervention and control districts of the study

Malaria is endemic in Mozambique, with a prevalence of 40.2% in children between 6 and 59 months of age [2]. The central and northern provinces of Zambezia and Nampula have the highest prevalence (67.9 and 66.0%, respectively), and the southern provinces of Maputo province and Maputo city have the lowest prevalence (2.8 and 2.2%, respectively) [2]. Manica province has a prevalence of 25.5% [2] (Fig. 1).

The interventions and controls districts were selected based on the following pragmatic and matching criteria: (i) they would benefit from the LLIN UCC in the concerned period; (ii) have population size similarities (intervention 1 with control 1, and intervention 2 with control 2); (iii) they have similar numbers of LLINs allocated for distribution (intervention 1 with control 1, and intervention 2 with control 2); (iv) they have rural characteristics; and (v) they are districts in provinces with high malaria prevalence.

### Description of study setting

Mozambique is mostly a rural country, with an estimated 5,058,763 households having an average of 5.0 members per household [16] resulting in an estimated 25,293,815 inhabitants. The illiteracy rate is 44.9% and most prevalent in the rural area [16]. Sixty-eight percent of the population have easy access to a health facility, i.e., walking less than 30 min to reach a health facility. However, this access is lower in rural areas [16]. The major malaria burden in Mozambique is in the central and northern provinces of Zambezia and Nampula [2, 17]. Nampula province has an estimated 1,016,455 households with an average 4.8 members per household [16] resulting in an estimated 4,878,984 inhabitants.

### Description of the health intervention

The universal coverage LLINs campaign is a proven health intervention promoting increased access, ownership, and use of LLINs to reduce malaria morbidity and mortality [7–9]. The current LLIN campaign in Mozambique has several phases related to trainings at provincial and district level, household registration, and LLIN distribution.

### Description of the implementation strategies

Multifaceted implementation strategies were designed and improved stage-by-stage. These implementation strategies were divided into two components: core/essential components and additional/complementary components. Three core components were designed and tested in the intervention districts: (i) use of coupons during household registration phase; (ii) use of stickers to identify the registered households; and (iii) a new criterion for LLIN allocation (one LLIN for every two persons). These three implementation strategies were developed and tested in Stage I. Household registration in the previous model (control districts) was undertaken without the use of coupons or stickers to identify the registered households, and variables such as name, age, gender, and family relationship of household members were collected and later analysed regarding possible sleeping patterns. The sleeping patterns were the LLIN ascription criteria (Table 1).

**Table 1:** Specifications of the implementation strategy

Dimensions	Intervention (after) strategies	Control (before) strategies: previous distribution model
Actor(s)	Institutional (health professionals) and community volunteers (household registrars) actors that implemented the campaign. Civil society partners	
Action(s)	The health intervention: LLINs universal coverage campaign with the following new implementation strategies Core components: (i) coupons; (ii) stickers; and (iii) one LLIN for every two people ascription criterion Complementary components: (i) mapping and micro-planning (improvements added in Stage II); (ii) training; (iii) support supervision	The health intervention: LLINs universal coverage campaign with the following previous implementation strategies (i) allocation of LLIN is based on sleeping patterns according to data collected during household registration (age, sex, family relationship); (ii) number of LLINs per household known during distribution phase; (iii) distribution points known after household registration; (iv) training and support supervision during all campaign phases
Target(s) of the action	Health professionals and community volunteers (household registrars): knowledge and skills about the intervention	
Temporality	Stage I: October–December 2015: Gurue and Sussundenga districts Stage II: August to November 2016–Nampula province	Stage I: October–December 2015: Alto-Molocue and Machaze districts Stage II: not applicable^a^
Dose: measured in terms of duration, frequency, and coverage	Trainings duration and coverage: 10 days for micro-planning and training of trainers for implementation (5 members of district team), 8 h (1 day) for preparation of registrar trainers (1 registrar trainer per 15 household registrar), 16 h (2 days) for registrar training (assuming 1 registrar can register 20 households per day and 140 households in 7 days), 8 h (1 day) for training of distribution teams (5 members for each distribution team). Seven days for household registration. Five days for LLIN distribution Frequency: once	Trainings: 3 days for micro-planning, 4 h for preparation of registrar trainers, 4 h for registrar training (1 registrar trainer per 15 household registrar), 4 h for training of data analysts, 56 h (7 days) for analysis of household registration data, 8 h (1 day) for training of distribution teams. Seven (7) days for household registration. Five days for LLIN distribution Frequency: once
Implementation outcomes	Coverage-type: percentage of LLINs distributed; percentage of target households benefited	
Justification	Programmatic justification: the type of household registration, the complex criteria for LLIN attribution, and the long queues to benefit the LLINs related to the previous campaign strategy made it necessary to design the new implementation strategy Theoretical justification: Socio-ecological model embedded in social practice theory	Theoretical justification: Socio-ecological model. Working with institutional and community actors to achieve better health outcomes

^a^Stage II was implemented only with the new implementation strategies

Three complementary components were added and implemented: (i) mapping and micro-planning (improvements added in Stage II); (ii) training; and (iii) support supervisions. Table 1 summarizes the implementation strategies during Stage I and II.

### Coupons

**Fig. 2:**
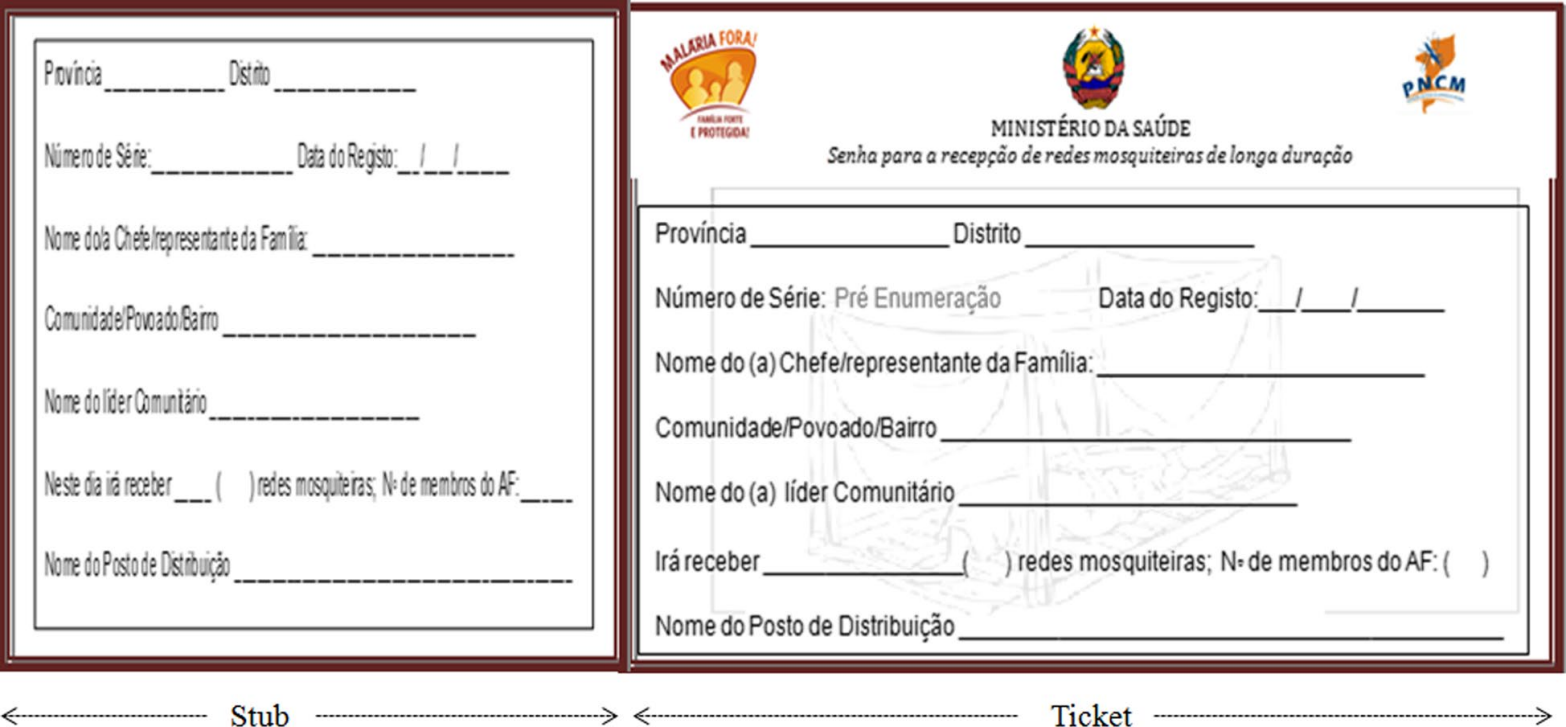
Coupon for household registration

The coupons have two parts, the stub, which is for control, and the ticket, which is given to the registered household members. Identical information appears on both the ticket and the stub. The coupon includes a single pre-enumeration area, background image (watermark) of a mosquito net, and information about the province, district, community, name of head of household, name of head of community, number of household members, number of LLIN to benefit, and the name of the distribution points (Fig. 2). The coupon is then later exchanged for the corresponding number of LLINs in the distribution phase.

### Sticker

**Fig. 3:**
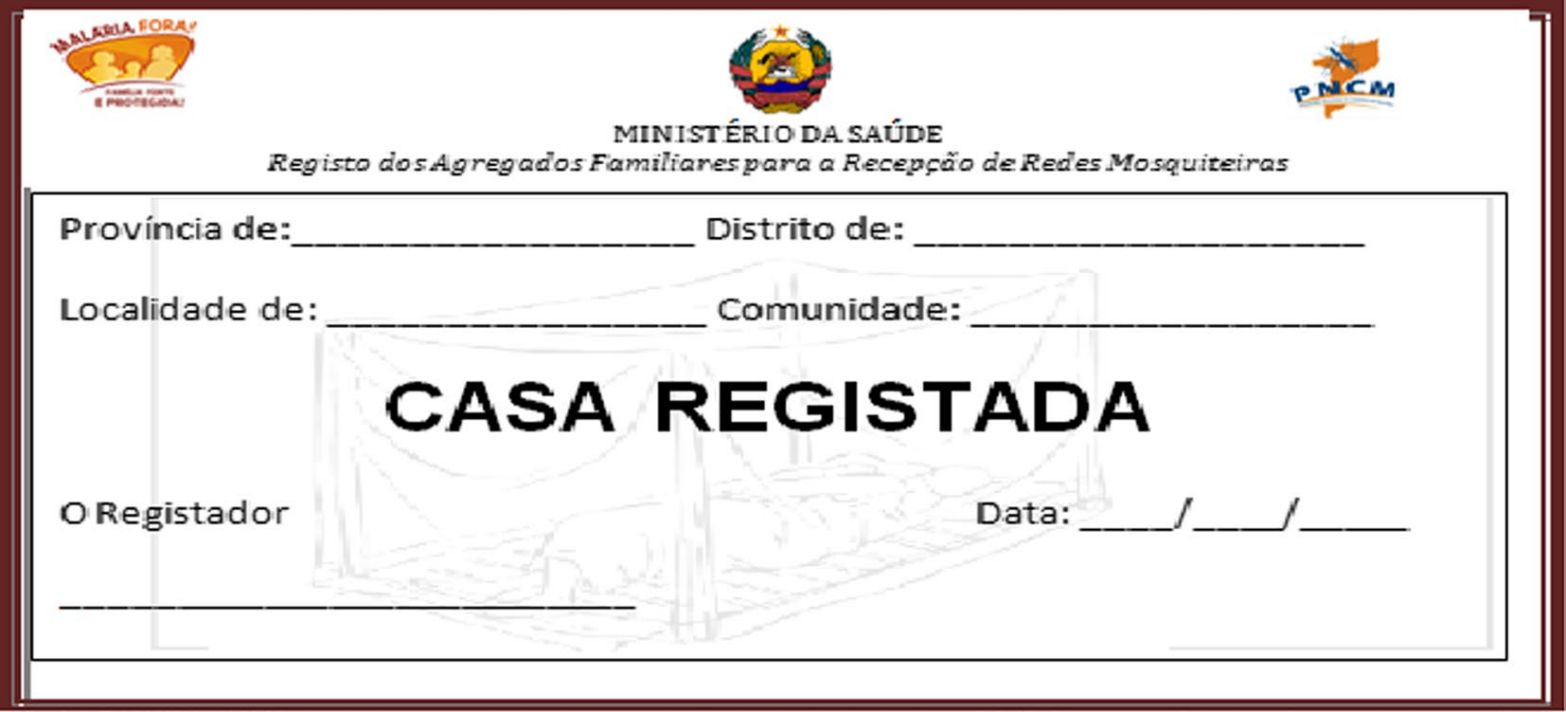
Stickers to identify the registered households

The sticker has information about the province, district, town, community, registration date, and the name of the registrar, along with a background image (watermark) of a mosquito net (Fig. 3).

### Long-lasting insecticidal nets ascription criterion

The LLIN ascription criterion was one LLIN for every two persons (rounded up in case of decimal number) with a maximum (cap) of four LLIN per household, i.e., families with nine or more household members received four LLINs. This LLIN capping criterion was established only in one district in Stage II.

### Mapping and micro-planning

Before mapping and micro-planning each district was given a list of data to be collected. This included population by each locality and administrative post, existing health and school infrastructures, sanctuaries, formal and informal markets, warehouses, distances map, roads, bridges, and remote zones of difficult access. This information is gathered onto a map that allows performing the micro-planning.

The micro-planning process uses a Microsoft office excel^®^ based tool with the following components: (i) position micro-plan; (ii) household registration plan; (iii) transport information; (iv) distribution plan; (v) transport plan; and (vi) crucial materials needed for registration and distribution. It is an iterative tool that allows easily identifying: (i) the population in each district by localities and communities; (ii) the warehouse that will serve each distribution point; (iii) number of LLINs and bales needed for each distribution point; (iv) number of household registrars needed for household registration process in a particular set of communities; (v) number and type of vehicles needed to transport LLINs from the main district warehouse to satellite warehouses (those located at community level) or distribution points; (vi) number of teams needed to distribute the LLINs in 5 days; and (vii) quantities of crucial materials needed for household registration and distribution phases. The tool is available as Additional file 1: Appendix 1.

### Training

Two training of trainers (ToT) actions were carried out at the central level. The first and second ToT were conducted between August and September 2016 in order to prepare all National Malaria Control Programme (NMCP) key staff and civil society partners, namely: World Vision International (WVI), Malaria Consortium, Food for the Hungry association, and Foundation for the Community Development.

A cascade of trainings followed the central level ToT, namely: (i) micro-planning and implementation ToT; (ii) training for household registration phase; and (iii) training of distribution teams and satellite warehouse keepers. During the micro-planning and implementation ToT (carried out at provincial level) several topics are covered, such as: (i) mapping process; (ii) micro-planning process; (iii) rationale of the new UCC implementation strategies; (iv) logistics (direct and reverse logistics related to LLINs); (v) household registration process (including quality control); (vi) communication aspects related to UCC; and (vii) LLIN distribution organization process.

### Support supervision

Support supervision is a critical implementation strategy that follows the mapping and micro-planning at provincial and district level. One provincial-level supervisor per district was assigned to support the UCC implementation. These supervisors (also called mentors) have the role of ensuring that all processes are carried out as planned (high fidelity) in their district. A structured supervision team was established at the district level and included: (i) a coordination group of five elements (district health team trained during micro-planning); and (ii) one supervisor for each 15 household registrars. Additionally, an action-checklist was developed in order to ensure appropriate support supervision process by central, provincial, and district supervisor teams.

### Outcomes and statistical analysis

A coverage-type implementation outcome was used as primary expected outcome in Stages I and II:

- Percentage of LLIN distributed calculated as number of distributed LLINs/number of planned LLINs × 100; the planned LLIN was determined by dividing the number of people by 1.78 (rounded up to 1.8) [18];
- Percentage of target households benefited calculated as number of households that received LLINs/number of registered households × 100.

All registered households were considered as the denominator for determining household coverage. Since it was a before-and-after design, odds ratio and 95% confidence interval were used to compare and establish differences between intervened and control districts. Implementation effectiveness was also calculated in Stage I to measure the extent of differences between intervention and control districts’ implementation outcomes. Implementation effectiveness measure was chosen as it reflects “effectiveness” in implementation studies, i.e., the equivalent of efficacy under “real world” implementation conditions. According to Gupta [19], implementation effectiveness or effectiveness at strategy implementation should be measured in the form of comparison between actual performance and a priori expectations rather than on an absolute scale.

- Implementation effectiveness = (outcome proportion with new implementation strategy—outcome proportion with previous implementation strategies) × 100/outcome proportion with previous implementation strategies.

The WINPEPI [20] version 11.60 computer programs were used for statistical analysis. For all statistical procedures, a 0.05 significance level was adopted for rejecting the null hypothesis.

In Stage II (Nampula province LLIN UCC implementation) only administrative data are reported using absolute frequency for registered households and distributed LLINs. Percentage of households benefited, and percentage of distributed LLIN were calculated. There were no control districts or provinces at this stage.

## Results

### Stage I

Household registration results revealed an existence of 136,985 and 120,446 households in the intervention and control districts, respectively. These household registration results also revealed a need for 344,770 and 284,873 LLINs in the intervention and control districts, respectively.

Nearly 88% (302,648) of planned LLINs were distributed in the intervention districts (Gurue and Sussundenga) compared to 77% (219,613) in the control districts (Alto Molocue and Machaze), with an implementation effectiveness of 12.2% [OR: 2.14 (95% CI 2.11–2.16; p < 0.001)]. Also, Stage I results revealed that 80.6% (110,453) of households received at least one LLIN in the intervention districts compared to 72.8% (87,636) households in the control districts with an implementation effectiveness of 9.8% [OR: 1.56 (95% CI 1.53–1.59); p < 0.001].

### Stage II

In Stage II (massive pilot in Nampula province) micro-planning figures revealed a total of 5,638,667 inhabitants and 1,282,512 households. After household registration these figures increased to a total of 7,038,427 inhabitants (24.8% increase) and 1,373,002 households (7.1% increase) (Table 2). During LLIN distribution several districts retrieved more coupons than what had been delivered (fake coupons). The most impressive case of this was in Mossuril district, where 47,231 out of 25,969 delivered coupons were retrieved (Table 2). Despite this, 3,536,839 LLINs (98.4% of the allocated LLINs) were delivered, covering 1,353,827 households (98.6% of the registered households).

**Table 2:** Population, households and coupons delivered and retrieved during Stage II

Districts	Population			Households			Coupons		
	After micro-planning	After HHR	%	After micro-planning	After HHR	%	Delivered	Retrieved	%
Angoche	446,064	612,491	37.3	83,049	107,960	30.0	107,890	84,049	77.9
Liupo	105,617	140,236	32.8	20,469	24,613	20.2	24,613	26,548	107.9
Mecuburi	197,275	208,387	5.6	46,851	49,001	4.6	49,131	44,942	91.5
Memba	295,251	385,672	30.6	66,504	73,291	10.2	73,341	66,855	91.2
Mogincual	113,717	146,330	28.7	21,220	25,904	22.1	25,953	21,699	83.6
Mogovolas	674,001	491,777	− 27.0	112,011	122,283	9.2	107,387	76,958	71.7
Moma	137,331	276,859	101.6	72,297	51,655	− 28.6	50,146	49,923	99.6
Murrupula	212,692	425,973	100.3	44,261	75,055	69.6	75,914	39,230	51.7
Nacaroa	150,588	206,577	37.2	31,753	41,400	30.4	40,714	33,663	82.7
Rapale	179,362	230,600	28.6	72,889	44,992	− 38.3	44,992	45,229	100.5
Ribaue	314,615	306,341	− 2.6	66,077	59,375	− 10.1	59,108	59,796	101.2
Nampula	746,638	789,361	5.7	159,633	155,854	− 2.4	155,854	160,094	102.7
Erati	329,681	472,101	43.2	79,140	94,714	19.7	94,583	89,977	95.1
Ilha Moz	55,890	94,071	68.3	13,973	15,361	9.9	15,361	14,380	93.6
Lalaua	79,341	149,018	87.8	22,206	27,184	22.4	27,184	27,873	102.5
Larde	83,967	126,257	50.4	20,630	22,504	9.1	22,504	20,806	92.5
Malema	197,836	234,028	18.3	49,459	48,864	− 1.2	48,864	49,630	101.6
Meconta	190,279	253,867	33.4	48,635	52,089	7.1	52,089	49,987	96.0
Monapo	402,534	556,372	38.2	95,612	116,503	21.8	115,197	109,665	95.2
Mossuril	178,671	143,044	− 19.9	35,020	25,969	− 25.8	25,969	47,231	181.9
Muecate	116,606	170,657	46.4	29,002	31,337	8.1	31,337	31,942	101.9
Nacala Porto	293,931	437,379	48.8	60,944	72,889	19.6	72,889	75,236	103.2
NacalaVelha	136,780	181,029	32.4	30,877	34,205	10.8	34,205	35,537	103.9
Total	5,638,667	7,038,427	24.8	1,282,512	1,373,002	7.1	1,355,225	1,261,250	93.1

*HHR* household registration

## Discussion

Stage I and II results shows that more LLINs were distributed and more households benefitted as a result of the new implementation strategies. This might be attributed to the coupons and sticker implementation strategy. The coupons seem to have had the desired effect, namely: (i) ensured the necessary confidence for the households that will in fact receive LLINs; (ii) identified the distribution point that households should go to in order to obtain LLINs; and (iii) facilitated confirmation that the household was registered, i.e., during LLINs distribution phase, the coupon was exchanged for LLINs. This chain of events, which constitutes a positive gradient of demand behaviour, is here termed as “the coupon effectˮ. This effect has also been observed in other studies in which coupons or vouchers were used [10–15]. Another explanation is that at the time of registering households a sticker was installed to identify registered houses. In this way, unregistered households were easily identified and registered, thereby ensuring that more households are registered and can benefit from LLINs. This additional factor is referred to herein as “the sticker effectˮ. The combination of these factors (coupon and sticker effect), which encourages the target households to obtain the LLINs and determines their greater ownership, constitute what will be called herein “the coupon-sticker effect”.

During a wrap up meeting held in early December 2016, with the main stakeholders involved in the Stage II massive pilot implementation, several weaknesses were reported, and can be summarized in three critical features: coordination, planning, and some loss of implementation fidelity.

Coordination at provincial and district levels were not well established, leading to incomplete readiness and UCC preparation. The planning process (the micro-planning tool) was not finalized, leading to deficiencies in the implementation process. Not establishing a cap (i.e. maximum LLIN per household) also led to inflation in the reporting of number of household members (population increase of 25% between micro-planning and after household registration figures without corresponding increase in households). This happened because the households realized that increasing the number of household members would benefit them with more LLINs. Another factor contributing to this inflation was the poor quality of the coupons, leading to coupon counterfeiting.

Implementation challenges are always present in the “real world”. The goal is not to eliminate the implementation challenges (a nearly impossible task) but to reduce them sufficiently to implement the strategy with high fidelity. Only by understanding and measuring whether an intervention has been implemented with fidelity can researchers and practitioners gain a better understanding of how and why an intervention works, and the extent to which outcomes can be improved [21]. In fact, some loss of fidelity was noted during the Stage II implementation process, resulting in several constraints, as reported in the results section. However, if a high fidelity level is not achievable, ensuring adequate adaptation of the strategy to fit the local context should at least be attained. In order to ensure effectiveness of the implementation, adaptation may occur with complementary components, but fidelity is mandatory for the core components, i.e., ensuring high quality availability of coupons and stickers, and ensuring application of the new formula (one LLIN for every two people with a cap of four LLINs per household). An additional and required adaptation of the strategies is the introduction of independent monitoring of the household registration, in order to promptly detect errors and correct them during this critical UCC phase.

## Conclusions

Stage I results showed greater availability and coverage of target households with LLIN in districts with the newly implemented strategies. The results of Stage II were also encouraging despite some problems related to population inflation and the quality of the coupons produced. Implementation strategies are important for reaching an effective health outcome. The importance of defining core and complementary components of a multifaceted implementation strategy resides in ensuring fidelity of the core components, and eventual adaptation of the complementary components to suit the local context. For the country-wide UCC some adaptation of the strategies is required, such as: ensuring high quality production of the coupons and stickers, capping the number of LLINs per household, and introducing independent monitoring of the household registration.

## Additional file

**Additional file 1: Appendix 1.** Micro-planning tool.

## Abbreviations

AMP: Alliance for Malaria Prevention
CI: confidence interval
HHR: household registration
LLIN: long-lasting insecticidal nets
NMCP: National Malaria Control Programme
OR: odds ratio
ToT: training of trainers
UCC: universal coverage campaign
WVI: World Vision International
