# Supplementary material for: Implementation strategies to increase access and demand of long-lasting insecticidal nets: a before-and-after study and scale-up process in Mozambique
Source: Malar J. 2017 Oct 25;16:429. doi: 10.1186/s12936-017-2086-3 (PMC5679509; doi:10.1186/s12936-017-2086-3)
Supplement: Supplementary file 1 — Additional file 1: Appendix 1. Micro-planning tool. [file 12936_2017_2086_MOESM1_ESM.pdf]

Microplanning tool for rural areas

District		insert the name

Population	0
Households	0
Number of bed nets	0
Number of household registrars	0

Number of Administrative Posts	

Number of satellites warehouses		Insert the number of satellites warehouses
Number of Headquarter distribution poir	0	
Total number of distribution points	0	
Number of distributors at headquarter	0	*Tem relação com a última célula da coluna Z (Z 1519)*
Total number of distributors	0	*Tem relação com a última célula da coluna Z (Z 1518)*
Number of localities		Insert the number

Logística de Materiais Cruciais da Campanha	
Number of household registration forms needs	0
Number of block of coupons needs	0
Number of rolls of stikers needs	0
Number of bed nets distribution form needs	#DIV/0!
Number of benefited households forms needs	#DIV/0!
Number of clipboards / backpacks needs	0

						4									20	7					2	750	5		
			Position Micro-Plan												Household registration plan				Transport information			Bed Nets Distribution phase			
No	Distribution Points (DP)	DP in headquarter? (Y/N)		villages/communities (Locality /Administrative Post)	Population	Households	Warehouse	Distance from main warehouse (km)	Distance from satellite warehouse to DP (km)	Distance from village/community to DP (km)	Number of bed nets for each DP	Number of bales for each DP	DP warehouse (m2 for height = 2m)	Access	Number of household registrar needs	Number of household registration days		household registrars teams	Viagens Camião pequeno @ 110 balões	Viagens Land Cruiser Pickup @ 24 fardos	Motorizadas e outro transporte	Numero de equipas (1 equipa = 5 Membros fixos)	Number of distribution days	Number of the team	Equipas que se Move
1						0					0	0.0	0.0			0.0			0.00	0.0	0	0.0	0.0		
						0										0.0									
						0										0.0									
						0										0.0									
						0										0.0									
						0										0.0									
						0										0.0									
						0										0.0									
						0										0.0									
						0										0.0									
						0										0.0									
						0										0.0									
						0										0.0									
					0	0									0	0.0									
